# Characterization of *Bacillus pacificus* G124 and Its Promoting Role in Plant Growth and Drought Tolerance

**DOI:** 10.3390/plants13202864

**Published:** 2024-10-13

**Authors:** Xiaolan Ma, Benyin Zhang, Xin Xiang, Wenjing Li, Jiao Li, Yang Li, Lam-Son Phan Tran, Hengxia Yin

**Affiliations:** 1 State Key Laboratory of Plateau Ecology and Agriculture, Qinghai University, Xining 810016, China; ys220713000143@qhu.edu.cn (X.M.); ys230713000152@qhu.edu.cn (W.L.); ys230713000135@qhu.edu.cn (J.L.); ys230860010516@qhu.edu.cn (Y.L.); 2 College of Eco-Environmental Engineering, Qinghai University, Xining 810016, China; benyinzhang@qhu.edu.cn (B.Z.); xiangxingogo@163.com (X.X.); 3 Department of Plant and Soil Science, Institute of Genomics for Crop Abiotic Stress Tolerance, Texas Tech University, Lubbock, TX 79409, USA

**Keywords:** *Bacillus pacificus*, drought tolerance, growth-promoting properties, antioxidant enzyme activities, osmolyte

## Abstract

Drought represents a major environmental threat to global agricultural productivity. Employing plant growth-promoting rhizobacteria (PGPR) offers a promising strategy to enhance plant growth and resilience under drought stress. In this study, the strain G124, isolated from the arid region of Qinghai, was characterized at the molecular level, and its ability to enhance plant drought tolerance was validated through pot experiments. The findings revealed that the strain G124 belongs to *Bacillus pacificus*, with a 99.93% sequence similarity with *B. pacificus* EB422 and clustered within the same clade. Further analysis indicated that the strain G124 demonstrated a variety of growth-promoting characteristics, including siderophore production, phosphate solubilization, and the synthesis of indole-3-acetic acid (IAA), among others. Moreover, inoculation with *B. pacificus* G124 resulted in significant enhancements in plant height, leaf area, chlorophyll content, relative water content, and root development in both *Arabidopsis thaliana* and *Medicago sativa* seedlings under drought conditions. Additionally, G124 boosted antioxidant enzyme activities and osmolyte accumulation, while reducing malondialdehyde (MDA) and reactive oxygen species (ROS) levels in *M. sativa* seedlings exposed to drought. These findings suggest that *B. pacificus* G124 holds significant promise for enhancing plant drought tolerance and could be effectively utilized in crop management strategies under arid conditions.

## 1. Introduction

Plants often confront unfavorable conditions during their growth in complex natural environments. Among these, drought is one of the most prevalent environmental stresses, significantly hampering crop growth and yield [1]. The acceleration of climate change and global warming is leading to more frequent and severe drought events, with projections indicating that over 50% of the planet will face water scarcity by 2050 [2]. This impending crisis, a ticking time bomb, underscores the critical need for sustainable solutions to enhance plant tolerance to drought.

A myriad of soil microorganisms influence plant growth, development, and stress tolerance [3]. Increasing evidence underscores the pivotal role of the rhizosphere-associated microbiota in plant health, growth, and development [4,5]. They are critical players in plant adaptation under diverse environmental growth conditions, with their composition being influenced by host and environmental factors [3]. Due to increased soil heterogeneity, nutrient mobility, and limited utility, drought significantly and negatively impacts microbial composition and structure, thereby exacerbating the stress effect on plants [6,7,8]. Several studies have found that specific microbes, including those in rhizosphere microbiota, can alleviate drought effects on host plants [9,10,11]. Conversely, in response to drought, host plants can alter the composition and structure of their associated rhizosphere microbiota, aiming at improving host environmental adaptation [12]. Therefore, altering the community structure of the rhizosphere microbiota is expected to be an effective strategy to improve the drought tolerance of host plants.

Plant growth-promoting rhizobacteria (PGPR) are those that colonize the inter- or intra-roots of plants. They directly or indirectly enhance plant growth and development under biotic and abiotic adversities by promoting nitrogen fixation, increasing nutrient uptake, improving soil properties, and inhibiting the multiplicity and action of plant pathogens [13,14,15]. A mounting body of evidence suggests that exploring PGPR–host interactions is an effective strategy to strengthen plant drought tolerance. For instance, PGPR have been shown to improve the tolerance of a variety of crops, including rice (*Oryza sativa*) [16], maize (*Zea mays*) [17], wheat (*Triticum aestivum*) [18], and barley (*Hordeum vulgare*) [19], under drought. These crops have demonstrated varying degrees of improvement in growth indexes, such as a more developed root system, increased aboveground biomass, improved photosynthesis, and reduced oxidative damage under drought conditions. The mechanisms by which PGPR mediate drought tolerance in plants are intricate and mainly include the alteration of root system structure, enhancement of antioxidant capacity, secretion of osmoregulatory substances [e.g., extracellular polysaccharide (EPS)], synthesis of phytohormones, such as growth hormone [indole-3-acetic acid (IAA)], abscisic acid (ABA), and gibberellic acid (GA), and the production of 1-aminocyclopropane-1-carboxylate (ACC) deaminase, as well as volatile organic compounds (VOCs) [20]. Therefore, the discovery of new PGPR resources is crucial for improving plant drought tolerance.

Microorganisms surviving in extremely arid habitats tend to be more tolerant under arid environments. They can be an essential source of PGPR for improving plant drought tolerance. For example, actinomycetes isolated from the inter-root soil of *Panicum turgidum* in the semi-arid environment of Saudi Arabia can mitigate the adverse effects of drought on maize growth and physiological performance [21]. In a previous phase of this study, the strain G124, isolated from the Gobi soil of Qinghai Province, exhibited potential plant growth-promoting and drought-resistance properties. Building on this foundation, the current research delves into molecular characterization, growth-promoting trait analysis, and pot experiments involving G124 to elucidate its taxonomic classification and the physiological and biochemical mechanisms underlying its enhancement of plant drought tolerance. This work will establish a basis for the future development and application of bacterial agents aimed at improving plant resistance to drought conditions.

## 2. Results

### 2.1. Morphological Observation of the Strain G124

**Figure 1:**
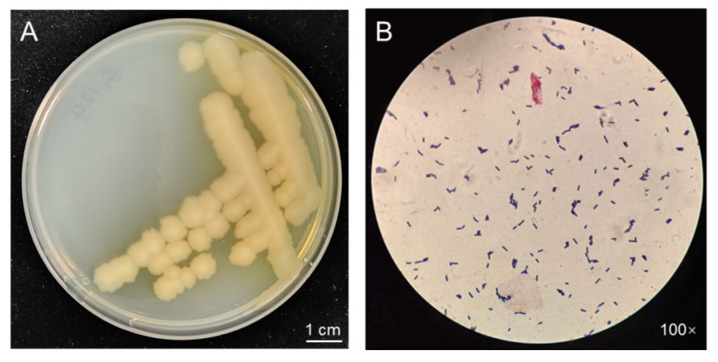
Morphology of strain G124 on LB plate (**A**), and Gram staining and microscopic observation (**B**).

When grown on Luria–Bertani (LB) medium for three days (Figure 1A), strain G124 presented intriguing characteristics. The yellowish-white, irregularly edged, wrinkled surface and non-smooth colonies were consistent with the morphological characteristics of *Bacillus* spp. Moreover, a single colony was selected for Gram staining and microscopic observation, revealing a purple stain and the distinct morphology of a rod-shaped bacterium. These observations confirmed the strain’s classification as a *Bacillus* sp. (Figure 1B).

### 2.2. Molecular Identification of the Strain G124

**Figure 2:**
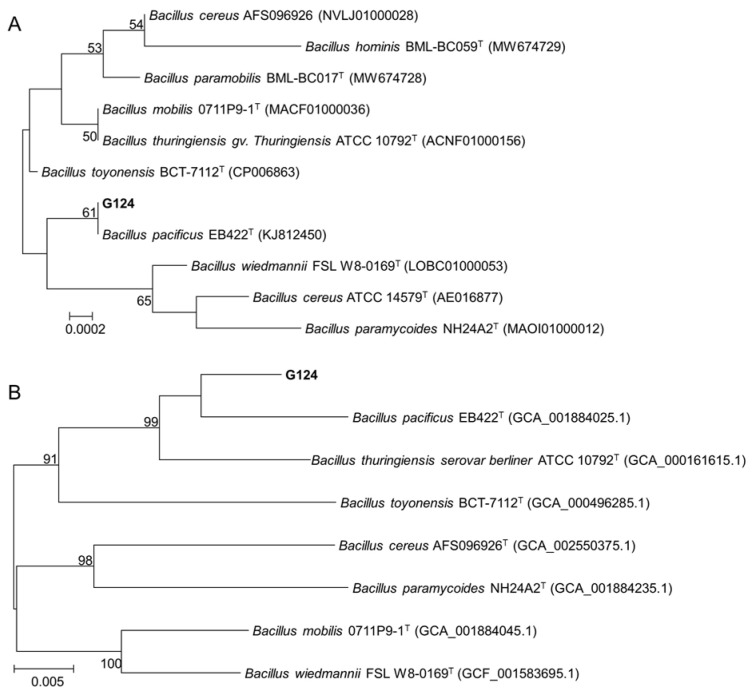
Phylogenetic trees constructed with 16S rDNA gene sequences (**A**) and concatenated housekeeping genes (**B**).

To further determine the taxonomic status of strain G124, we first obtained a sequence with a size of 1440 bp by amplifying and sequencing the 16S rDNA gene of strain G124. This sequence, which was deposited in the GenBank database (accession number PP806098), was then used to search for homology in the EzBioCloud database. The result showed a maximum similarity of 99.93% between the gene sequence and the 16S rDNA sequence of *B. pacificus* EB422. This finding was further reinforced by constructing a phylogenetic tree using MEGA 11.0 (Figure 2A), which revealed that strain G124 belonged to the genus *Bacillus* and clustered with *B. pacificus* EB422 in the same clade. This led us to rename the strain to *B. pacificus* G124. The phylogenetic tree of six concatenated housekeeping genes also confirmed the significance of these findings (Figure 2B).

### 2.3. Potential Host Growth-Promoting Properties of the Strain G124

**Figure 3:**
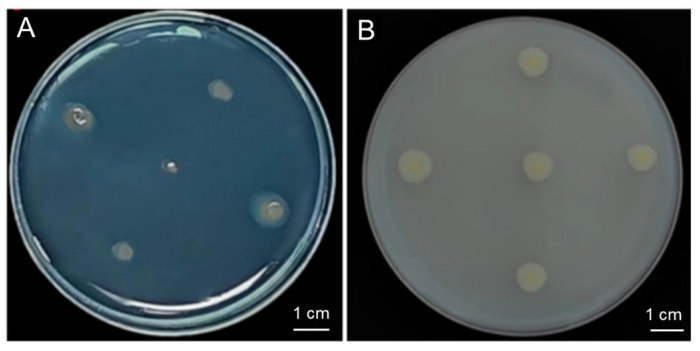
Siderophore-producing (**A**) and phosphorus-solubilizing capacity (**B**) of *B. pacificus* G124.

The strain G124 produced an orange-yellow halo circle on the chrome azurol S (CAS) plate, and the D/d mean ratio was determined to be 1.90, indicating a strain with a high capacity of siderophore production (Figure 3A and Table 1). Additionally, strain G124 produced an obvious phosphorus-solubilizing circle on the Pikovskaya (PKO) medium, and the D/d mean ratio was 0.59, indicating a strong phosphorus solubilization capacity (Figure 3B and Table 1). The IAA-producing ability of strain G124 was also measured, and its secretion was found to be 0.32 μg·mg^−1^. In addition, strain G124 produced EPSs, with an EPS concentration of 1.24 mg·mg^−1^, and the ACC deaminase, with an activity of 0.054 U·mg^−1^ (Table 1). These indicators together suggest that strain G124 has the potential for promoting the growth and drought tolerance of its host.

### 2.4. B. pacificus G124 Improves Growth and Physiological Parameters of Arabidopsis Thaliana and Medicago Sativa Plants under Drought

**Figure 4:**
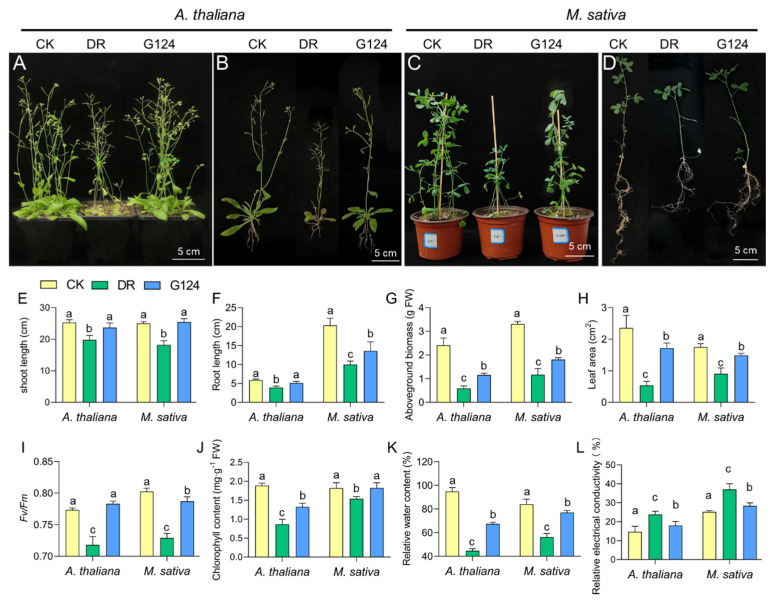
Effect of *B. pacificus* G124 inoculation on the phenotype and physiological parameters of *A. thaliana and M. sativa.* seedlings under drought stress. CK, normal watering group; DR, non-inoculated and drought-stressed group; G124, G124-inoculated and drought-stressed group. (**A**,**B**) the effects of strain G124 on the growth and root phenotypes of *A. thaliana* under drought stress; (**C**,**D**) the effects of strain G124 on the growth and root phenotypes of *M. sativa* under drought conditions. (**E**–**L**) a comparative analysis of shoot length (**E**), root length (**F**), aboveground biomass (**G**), leaf area (**H**), maximum photochemical efficiency (**I**), chlorophyll content (**J**), relative water content (**K**), and relative electrical conductivity (**L**) across different treatment groups for both *A. thaliana* and *M. sativa*, respectively. The different lowercase letters above the error bars indicate statistically significant differences between treatments (*p* < 0.05).

To evaluate whether *B. pacificus* G124 could alleviate drought stress in plants, *B. pacificus* G124 culture was used to inoculate *A. thaliana* and *M. sativa* plants. In comparison with the CK group, both *A. thaliana* and *M. sativa* plants of the non-inoculated DR group presented shorter shoot and root lengths, while those of the *B. pacificus* G124-inoculated group showed significantly longer shoot and root lengths under drought stress (Figure 4A–D). Specifically, under drought stress, shoot and root lengths exhibited significant increases of 15.7% and 27.7% in G124-inoculated *A. thaliana* and 33.4% and 36.1% in G124-inoculated *M. sativa*, respectively, relative to their counterparts in the DR group (Figure 4E,F). Notably, the shoot lengths of drought-stressed seedlings from both species were recovered by *B. pacificus* G124 treatment to levels comparable to those of the respective CK group (Figure 4E). Additionally, the fresh aboveground biomass and leaf area of drought-stressed *A. thaliana* and *M. sativa* plants significantly decreased when compared with those of plants grown under normal conditions. Importantly, the fresh aboveground biomass of G124-inoculated *A. thaliana* and *M. sativa* plants showed substantial increases of 97.3% and 54.9%, respectively (Figure 4G), and the leaf area of the G124-group plants expanded considerably, by 219.2% for *A. thaliana* and 64.6% for *M. sativa*, relative to the respective values of DR seedlings during drought (Figure 4H). G124 seedlings in the CK and G124-inoculated groups exhibited a deeper green leaf coloration than DR seedlings under drought stress (Figure 4A–D), and further investigations into chlorophyll content and the maximum photochemical efficiency (*Fv*/*Fm*) showed that these two indicators of plants grown under drought remarkably declined in comparison with the CK and G124-inoculated groups. Furthermore, the results revealed substantial accumulations of chlorophyll content in G124-inoculated *A. thaliana* (by 46.2%) and *M. sativa* (by 16.2%) compared with the respective DR counterparts under drought. Additionally, the *Fv*/*Fm* was markedly improved in the G124-inoculated groups (Figure 4I,J), underscoring the positive impact of *B. pacificus* G124 inoculation on the photosynthetic capabilities of *A. thaliana* and *M. sativa* seedlings under drought conditions.

Drought stress featured a precipitous decline in the relative water content of seedling leaves, coupled with a pronounced increase in relative electrical conductivity. Indeed, our investigation also showed that the DR group plants of both species showed increased relative water contents and relative electrical conductivity when compared with the respective CK group (Figure 4K,L). In comparison with non-inoculated seedlings, however, inoculation with *B. pacificus* G124 facilitated the recovery of the relative water content in *A. thaliana* and *M. sativa* leaves, and resulted in 25.1% and 30.6% lower relative electrical conductivity, respectively, which showed a restoration to the level of the CK groups, under drought stress (Figure 4K,L). These findings suggested that *B. pacificus* G124 inoculation alleviated the negative effects of drought stress on *A. thaliana* and *M. sativa* plants.

### 2.5. B. pacificus G124 Improves Biochemical Characteristics of M. sativa Plants under Drought

**Figure 5:**
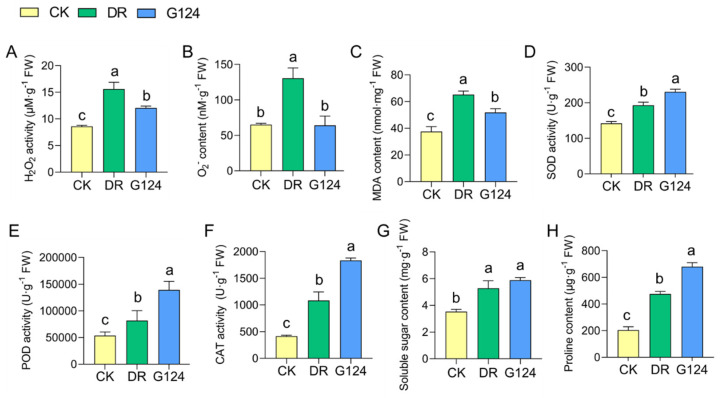
Improving effect of *B*. *pacificus* G124 on biochemical characteristics of *M. sativa.* plants exposed to drought. CK, normal watering group; DR, non-inoculated and drought-stressed group; G124, G124-inoculated and drought-stressed group. A–H, a comparative analysis of peroxides H_2_O_2_ (**A**), O_2_^−^ (**B**) and MDA (**C**) contents, antioxidant enzyme SOD (**D**), POD (**E**) and CAT (**F**) activities, and osmolyte soluble sugars (**G**) and proline (**H**) levels across different treatment groups for *M. sativa*, respectively. The different lowercase letters above the error bars indicate statistically significant differences between treatments (*p* < 0.05).

To elucidate the biochemically adaptive response of *B. pacificus* G124-inoculated plants to drought stress, *M. sativa* plants were further investigated. The concentrations of reactive oxygen species (ROS) like hydrogen peroxide (H_2_O_2_), superoxide anion (O_2_^−^) and malondialdehyde (MDA), antioxidant enzyme activities, and osmoregulatory substance levels in *M. sativa* plants across various treatment groups were assessed (Figure 5). Seedlings subjected to drought stress exhibited markedly increased levels of H_2_O_2_, O_2_^−^, and MDA compared with the CK group, signifying elevated oxidative stress. In comparison with CK plants, *B. pacificus* G124 inoculation significantly diminished H_2_O_2_, O_2_^−^, and MDA levels by 22.8%, 50.8%, and 20.5%, respectively (Figure 5A–C), suggesting an amelioration of drought-induced oxidative damage. Moreover, an upsurge in superoxide dismutase (SOD), peroxidase (POD), and catalase (CAT) activities was observed in DR seedlings experiencing drought when compared with CK seedlings, indicating that the antioxidant enzymatic system serves as a critical defense against oxidative stress. The activities of SOD, POD, and CAT were further increased in G124 plants by 62.4%, 158.7%, and 341.8%, respectively, compared with DR plants, upon G124 inoculation (Figure 5D–F), which may be an essential reason for the lower levels of ROS and MDA in the leaves of G124 plants than in those of DR plants. Additionally, drought stress promoted the biosynthesis and accumulation of soluble sugars and proline in the plants of the DR group relative to those of the CK group, imperative for osmoregulation, while *B. pacificus* G124 inoculation yielded even higher increments of these osmolytes of 66.5% and 234.2%, respectively, in G124 plants compared with those in the DR group (Figure 5G,H). Collectively, *B. pacificus* G124 inoculation modulated both antioxidant defense and osmoregulation in *M. sativa*, thereby mitigating drought-induced damage and enhancing plant drought resilience.

## 3. Discussion

Microorganisms in soil play a vital role in sustaining the stability and productivity of crops, as well as the well-being of natural ecosystems [22]. Advancements in microbial fertilizers are pivotal for fostering sustainable agricultural practices [23]. The identification and application of PGPR have recently emerged as a research focus due to their potential to safely and efficiently bolster crop yield and quality, while mitigating environmental impact and enhancing stress resistance [24]. In this study, we successfully isolated strain G124 from an arid soil environment and identified it as *B. pacificus* after thorough morphological and molecular analyses. *Bacillus* spp., particularly PGPR, are renowned for their ability to promote growth and yield and fortify plants against various environmental stressors [25]. Through comprehensive phylogenetic comparisons of 16S rRNA sequences and additional verification using six conserved housekeeping genes within the *Bacillus* genus, we established the accurate taxonomic identification of strain G124, thus setting a foundation for its future application.

Strain G124 exhibits robust growth-promoting properties, capable of synthesizing IAA, solubilizing inorganic phosphorus, producing siderophores, secreting EPSs, and demonstrating ACC deaminase activity (Figure 3 and Table 1). These traits not only aid plant growth but may also confer a capacity to plants to withstand adverse drought conditions [26]. For example, the IAA growth factor secreted by PGPR may contribute to the promotion of plant growth by promoting the elongation and growth of the cells at a relatively low concentration [27]. Phosphorus, being an essential nutrient, enhances plant immunity and resilience to various biotic and abiotic stressors, such as drought, pests, and pathogens [28]. Our results showed that strain G124 was able to solubilize phosphorus (Figure 3B and Table 1), and increasing the utilization of insoluble forms of macronutrients like phosphorus is an important property of PGPR for increasing crop yield and growth [29,30]. For example, inoculation of plants with highly efficient phosphorus-solubilizing bacteria significantly increased their phosphorus uptake and utilization [31]. EPSs are high-molecular-weight polymers composed of sugar groups [32]. Under drought conditions, EPS-producing bacteria not only improve soil quality by increasing water holding capacity but also protect plants from abiotic stresses [33]. EPS production by G124 probably enhances soil water retention and ultimately bolsters plant tolerance to drought. In a previous study, the EPS-producing PGPR strains *Proteus penneri*, *Pseudomonas aeruginosa*, and *Alcaligenes faecalis* showed increased plant biomass, root and stem lengths, and leaf area [34]. Similarly, siderophore-producing PGPR enhance iron availability in the soil, improving plant iron nutrition under stress conditions [35]. Another salient feature of G124 is its strong ACC deaminase activity (Table 1), likely suggesting that this strain has the capacity to alleviate the negative effect of drought stress on plants. This enzyme degrades the ethylene precursor ACC, thereby reducing the detrimental impact of ethylene on plant growth during stress, and thus bolstering drought resilience [36]. For example, Murali et al. (2021) conducted a screening of numerous bacteria exhibiting ACC deaminase activities from rhizosphere soil samples of pearl millet (*Pennisetum glaucum*), employing this activity as a biomarker. The authors finally isolated a *Bacillus* sp., namely a *B. amyloliquefaciens* strain, which displayed the most pronounced ACC deaminase activity and demonstrated a notable ability to enhance drought tolerance in *P. glaucum* [37].

Drought stress is known to significantly inhibit plant growth. Our research showed that drought markedly reduced the growth parameters of both *A. thaliana* and *M. sativa*, such as their shoot and root lengths, leaf area, and aboveground biomass. However, their inoculation with *B. pacificus* G124 partially reversed these damages (Figure 4E–H), which is in line with the findings on the drought mitigation effect of *Bacillus* spp. on fenugreek (*Trigonella foenum-graecum*) [38] and dwarf bamboo (*Fargesia rufa*) [39]. Additionally, the relative water contents of the tested plants, which is an indicator of plant status under drought, were restored almost to the control levels upon their inoculation with strain G124 (Figure 4K). Our finding was supported by the results of a study conducted with inoculated wheat plants under drought stress [33]. Leaf chlorophyll content is one of the most critical determinants of photosynthetic rate and biomass production [40]. In this study, chlorophyll content and fluorescence parameters of *A. thaliana* and *M. sativa* inoculated with *B. pacificus* G124 were significantly higher than the non-inoculated counterparts under drought stress (Figure 4I,J), suggesting that the inoculation of *B. pacificus* G124 could reduce the drought-induced damage to the chloroplast structure of *M. sativa* seedlings, contributing to maintaining their photosynthetic performance [41]. In addition, relative electrical conductivity is often related to the integrity of plant cells under stress conditions. When subjected to drought stress, plant cells are damaged, resulting in reduced integrity, which in turn causes an increase in relative electrical conductivity [42]. In this study, the relative electrical conductivity of *A. thaliana* and *M. sativa* seedlings inoculated with *B. pacificus* G124 was significantly lower than that of seedlings under drought conditions (Figure 4L), proposing that the inoculation of *B. pacificus* G124 mitigated cellular damage in inoculated plants, underscoring the G124’s role in improving drought tolerance. Collectively, the inoculation of *A. thaliana* and *M. sativa* plants with *B. pacificus* G124 significantly alleviated their drought-induced growth inhibition. Our findings also warrant further research to assess its ameliorative potential on other plant species exposed to drought.

To explore the impact of strain G124 on the improvement of biochemical properties of plants experiencing drought stress, we further analyzed several representative parameters related to biochemical responses in *M. sativa* plants grown under drought with and without G124 inoculation. When confronted with drought, plants frequently generate ROS, such as H_2_O_2_ and O_2_^−^, in chloroplasts, peroxisomes, and mitochondria, leading to severe cellular damage, including lipid peroxidation, DNA damage, and protein oxidation, ultimately disrupting the physiological and metabolic functions of plants [43]. In this study, drought stress led to augmented levels of H_2_O_2_ and O_2_^−^ in *M. sativa* seedlings compared with the control group (Figure 5A,B), indicating that the seedlings suffered a heightened oxidative stress triggered by drought. The increase in the MDA level (Figure 5C) also suggests that the degree of membrane lipid peroxidation is more severe, as MDA is often one of the most critical indicators characterizing the degree of cellular damage [44]. When subjected to oxidative stress, a plant’s antioxidant defense system will be activated to scavenge excess ROS, protecting plant cells from oxidative damage [45]. The results of the present study clearly showed that drought treatment significantly increased the activities of several antioxidant enzymes, such as SOD, POD, and CAT (Figure 5D–F), in *M. sativa*, which is a biochemical response as also observed in other plant species [46,47,48]. Intriguingly, *M. sativa* plants in the *B. pacificus* G124-inoculated group maintained higher levels of antioxidant enzyme activities and had significantly lower levels of ROS and MDA than those in the G124-untreated group under drought, suggesting that the enhancement of plant drought tolerance by *B. pacificus* G124 was associated with its ability to help increase the antioxidant enzyme activities to scavenge excessive drought-induced ROS from the drought-stressed plants [49,50].

Additionally, drought-stricken plants often accumulate phase-soluble substances in the cytoplasm, especially soluble sugars and proline, to maintain the osmotic pressure between the vesicle and its surroundings [51]. Besides its role in osmoregulation, proline may be the primary source of energy and nitrogen in the metabolic processes of plants under drought stress [52]. This study found higher soluble sugar and proline levels in *M. sativa* plants inoculated with *B. pacificus* G124 than in non-inoculated plants under drought stress. The soluble sugar and proline contents were increased by 68.44% and 189.39%, respectively, in the G124 group compared with the DR group (Figure 5G,H), strengthening the theory that producing more soluble sugars and proline in plants is a drought-responsive defense response.

## 4. Materials and Methods

### 4.1. Strain G124 and Plant Materials

Strain G124 was isolated from the arid habitat of Haixi, Qinghai, and kept in the State Key Laboratory of Plateau Ecology and Agriculture, Qinghai University, China. Plants used in inoculation were *A. thaliana* (Col-0) and *M. sativa*.

### 4.2. Morphological and Molecular Identification of G124 Strain

The morphological observation of strain G124 was carried out according to the *Berger Manual of Bacterial Identification* (8th edition). The strain was streaked on LB solid medium. The morphological characteristics of colonies were observed and recorded. These characteristics included color, shape, colony size, transparency, glossiness, texture, colony edge characteristics, and bulge shape. Subsequently, microscopic observation was performed after Gram staining. For molecular identification, the total DNA of strain G124 was extracted using SDS-CTAB, which was then used as a template for PCR amplification of the 16S rRNA gene using universal primers (Table S1). The purified PCR products were sent to the GENEWIZ company (Suzhou, China) for sequencing. The spliced sequences were then submitted to the online tool “Identify” (https://www.ezbiocloud.net/identify (accessed on 16 April 2024) in the EzBioCloud database for homologous sequence comparison. This comparison helped us find model strains that had higher sequence homology with the tested strains. The 16S rRNA genes of similar strains were then downloaded. These sequences were subjected to construct a neighbor-joining (NJ) phylogenetic tree using MEGA 11.0 (https://www.megasoftware.net/ (accessed on 16 April 2024)) with a self-expansion value of 1000. In addition, the sequences of six housekeeping genes, *triosephosphate isomerase* (*tpi*), *dihydroxy-acid dehydratase* (*ilvD*), *glycerol uptake facilitator protein* (*glp*), *pyruvate carboxylase* (*pycA*), *guanylate kinase* (*gmk*), and *phosphate acetyltransferase* (*pta*), of the *Bacillus* genus (Table S1) were amplified, sequenced, and tandemly spliced, for the construction of another NJ phylogenetic tree using the same approach.

### 4.3. Determination of Growth-Promoting Ability of G124 Strain

#### 4.3.1. Siderophore Production

The G124 strain was inoculated into 3 mL Tryptic Soy Broth (TSB) liquid medium with a 1% volume ratio for activation, cultured at 28 °C with shaking (180 rpm) until the OD_600_ reached about 1.0, and then spotted on the CAS test plate and cultured at a constant temperature of 28 °C for 72 h. Orange-yellow halo rings appearing around the colonies indicated the production of siderophore. Colony counter (icount20, Shineso science and technology Co., Ltd., Hangzhou, China) was then used to measure the diameter (D) of the transparent halo circle of the single colony and the diameter (d) of the colony. The ratio of D/d was used to evaluate the ability of siderophore production.

#### 4.3.2. Phosphorus-Solubilizing Capacity

The activated bacterium was spot-inoculated with inorganic phosphorus medium in three replicates and cultured at 28 °C for 72 h following the phosphorus-solubilizing circle method [53]. The phosphorus-solubilizing circle was observed, and its diameter (D) and colony diameter (d) were measured to determine the phosphorus-solubilizing ability.

#### 4.3.3. IAA Secretion Capacity

The strain G124 was inoculated in a TSB liquid medium containing tryptophan (100 mg·L^−1^). The 2 mL supernatant was centrifuged after 48 h of oscillation, and 50 μL of orthophosphoric acid (83% by volume) and 4 mL of Salkowski’s reagent (prepared with 50 mL H_2_O, 1 mL 0.5 mol·L^−1^ FeCl_3_, 30 mL 98% H_2_SO_4_) were added. The production of IAA was indicated by the pink color of the solution and determined by reading the absorbance at OD_530_. The standard curve was created using the uninoculated medium as a blank and IAA standard solutions (Figure S1).

#### 4.3.4. EPS Production Capacity

The crude polysaccharides were extracted, and the glucose standard curve was plotted using the phenol–sulfuric acid method (Figure S2) [54]. The absorbance was measured at 490 nm, and the measured absorbance was brought into the standard curve-fitting equation to obtain the polysaccharide concentration.

#### 4.3.5. ACC Deaminase Activity

The strain G124 was incubated with shaking for 12 h, collected by centrifugation, and resuspended in Dworkin and Foster (DF) medium without a nitrogen source (purchased from yuanye Bio-Technology Co., Ltd., Shanghai, China) by adding filter-sterilized ACC solution (0.5 M). Subsequently, the mixture was incubated with shaking for 24 h to induce the deaminase activity of ACC in the strain, and the G124 was collected and resuspended in Tris-HCl buffer (pH = 7.6). The product was measured by using 2,4-dinitrophenylhydrazine as a dye. α-ketobutyric acid was used as an assay to determine the ACC deaminase viability of the strain. The protein content of the bacterial suspension was determined using the Bradford method [55] with a standard sample of bovine serum albumin (BSA) as a control (Figure S3). ACC deaminase activity was measured as the amount of α-ketobutyric acid produced per unit of protein amount of the organism per unit of time. The amount of α-ketobutyric acid produced at 1 μM per minute was taken as one unit of enzyme activity (U), and the ratio between the unit of enzyme activity and the total protein content was regarded as ACC deaminase activity (U·mg^−1^) [56].

### 4.4. Preparation and Inoculation of G124 Suspension

Strain G124 was inoculated in TSB medium and cultured at 28 °C with shaking (180 rpm) until the OD_600_ value reached about 1.0. The culture was centrifuged to remove the supernatant and then resuspended in sterilized ddH_2_O to obtain the bacterial suspension for inoculation. In the pot test using *A. thaliana*, *A. thaliana* seeds were sterilized and sown on plates (10 × 10 cm; 70 seeds per plate) containing 1/2 MS medium (purchased from Solarbio Science & Technology Co., Ltd., Beijing, China). The plates were placed in an artificial climate chamber after stratification for 12 h at 4 °C for germination in conditions of 16 h light (with light intensity of 8000 Lux)/8 h dark and a temperature of 22–25 °C. After 7 days, *A. thaliana* seedlings with consistent growth were transplanted into pots (5 seedlings in each pot with the size of 8 × 8 × 7 cm) with 70 g soil per pot for bacterial inoculation.

In the pot test using *M. sativa*, sterilized *M. sativa* seeds were placed in Petri dishes (a diameter of 9 cm) with moist filter paper for germination. The culture conditions were consistent with that of *A. thaliana*. The seedlings with consistent growth were transplanted into pots (5 seedlings in each pot with a diameter of 10 cm and height of 9 cm) with 100 g soil per pot after 4 days of germination and grown for 20 days. Seedlings of both plant species were watered every 3 days with 30 mL water per pot each time. The soils for the *A. thaliana* and *M. sativa* planting were a 1:1 mixture of peat soil and vermiculite and a 1:1:1 mixture of sand, peat soil, and vermiculite, respectively. Both soil mixtures were unsterilized.

The pot experiment was divided into three groups (Table 2): the normal watering group (CK), the non-inoculated and drought-stressed group (DR), and the G124-inoculated and drought-stressed group (G124). After *A. thaliana* and *M. sativa* plants were grown in pots for 7 days and 20 days, respectively, they were inoculated with G124 culture. Inoculation was performed as root inoculation by pouring 1 mL of bacterial suspension around the root of each *A. thaliana* plant and 2 mL of bacterial suspension around the root of each *M. sativa* plant three times on three consecutive days. The corresponding volume of distilled water was added to each plant in the DR group in the same manner and time. For drought treatment, plants of the DR and G124 groups were not watered for two weeks.

### 4.5. Determination of Physiological and Biochemical Indices of Seedling Growth

The leaves (same positions) and root lengths of plants in different treatment groups were photographed, and the pictures were imported into Image J to calculate leaf area and root length, while plant height was measured with a tape measure. The *Fv*/*Fm* of the same tissue part of leaves in different treatment groups was measured by chlorophyll fluorescence meter (FluorPen FP110, Photon Systems Instruments, Drásov, Czech Republic) after the dark adaptation period of 30 min. The chlorophyll content, relative electrical conductivity, and relative water content of leaves were measured following the previous methods [17,40,57]. To determine the biochemical indexes in *M. sativa*, the leaves of *M. sativa* seedlings were collected and fully ground in liquid nitrogen. The activities of SOD, POD, CAT, and contents of H_2_O_2_, O_2_^−^, MDA, soluble sugars, and proline were determined according to the manual instructions using the respective reagent kits (Keming Bio-technology Co., Ltd., Suzhou, China).

### 4.6. Data Processing

Data were subjected to a one-way ANOVA using the software SPSS 26.0 to evaluate the variation significance among different groups. Then, Graphpad Prime 8.0 (https://www.graphpad.com/features (accessed on accessed on 7 May 2024)) was used to graph the experimental results. The data are presented as means and standard deviations.

## 5. Conclusions

This study molecularly characterized *B. pacificus* G124, isolated from Gobi soil in Qinghai, and evaluated its potential to enhance plant drought tolerance. The results demonstrate that the strain G124 not only produces siderophores and EPSs, solubilizes phosphate, synthesizes IAA, and exhibits ACC deaminase activity, but also significantly enhances plant height, leaf area, chlorophyll content, relative water content, and root growth and development in *A. thaliana* and *M. sativa*. Furthermore, the strain G124 mitigates drought-induced oxidative stress in *M. sativa* by enhancing antioxidant enzyme activity and reducing peroxides while maintaining cellular osmotic balance under drought conditions through increased accumulation of osmotic regulators. Future research will focus on the molecular mechanisms by which this strain modulates plant drought resistance. These findings suggest that strain G124 is a promising candidate for use in the development of drought-resistant biofertilizers.

## Figures and Tables

**Table 1:** Determination of growth-promoting ability of *B. pacificus* G124.

Strains	Phosphorus Solubilizing	Siderophore Production	IAA Content (ΜG·mg^−1^)	EPS Content (Mg·mg^−1^)	ACC Activity (U·mg^−1^)
Control	—	—	—	—	—
G124	+	++	0.319	1.238	0.054

++, stronger; +, strong; —, insignificant.

**Table 2:** Group designs of different treatments.

Group	*A. thaliana/M. sativa*
CK	Normal watering
DR	Injected with 1 mL/2 mL of distilled water
G124	Inoculated with 1 mL/2 mL of G124 strain suspension

## Data Availability

The raw data supporting the conclusions of this article will be made available by the authors on request.

## Supplementary Materials

The following supporting information can be downloaded at: https://www.mdpi.com/article/10.3390/plants13202864/s1, Figure S1: Standard curve for indole-3-acetic acid (IAA) assay; Figure S2: Glucose standard curve; Figure S3: Standard curves for determining α-ketobutyric acid (A) and protein contents (B); Table S1: Primers used in the present study.
