# Supplementary material for: Characterization of Bacillus pacificus G124 and Its Promoting Role in Plant Growth and Drought Tolerance
Source: Plants (Basel). 2024 Oct 13;13(20):2864. doi: 10.3390/plants13202864 (PMC11511372; doi:10.3390/plants13202864)
Supplement: Supplementary file 1 [file plants-13-02864-s001.zip › plants-3228168-supplementary.pdf]

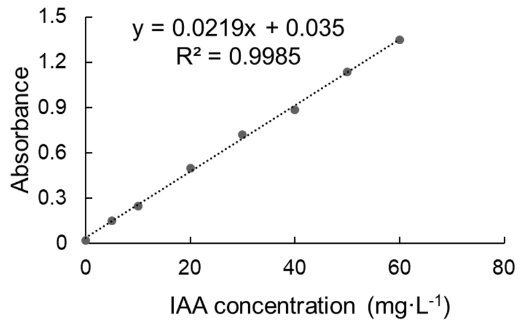

Figure S1 Standard curve for indole-3-acetic acid (IAA) assay.

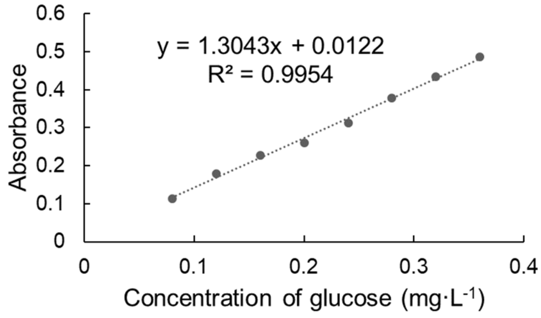

Figure S2 Glucose standard curve.

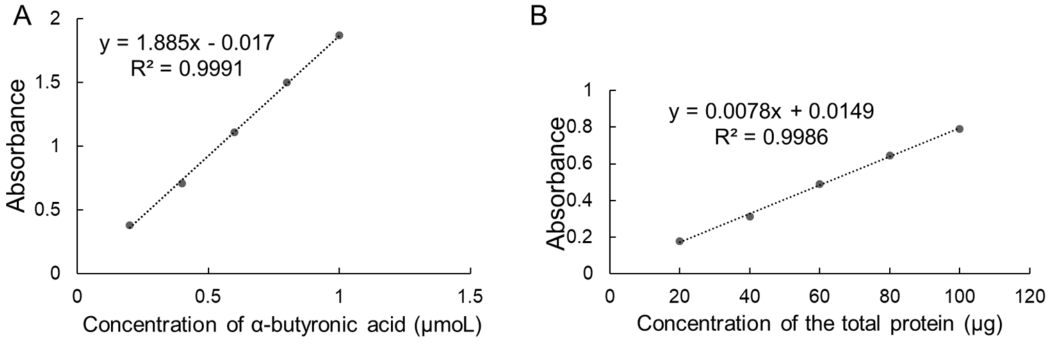

Figure S3 Standard curves for determining α-ketobutyric acid (A) and protein contents (B).

Table S1 Primers used in the present study.

Gene names		Primers（5'-3'）
16S rRNA	Forward	AGAGTTTGATCCTGGCTCAG (27F)
	Reverse	GGTTACCTTGTTACGACTT (1492R)
glp	Forward	GCGTTTGTGCTGGTGTAAGT
	Reverse	CTGCAATCGGAAGGAAGAAG
gmk	Forward	ATTTAAGTGAGGAAGGGTAGG
	Reverse	GCAATGTTCACCAACCACAA
ilvD	Forward	CGGGGCAAACATTAAGAGAA
	Reverse	GGTTCTGGTCGTTTCCATTC
pta	Forward	GCAGAGCGTTTAGCAAAAGAA
	Reverse	TGCAATGCGAGTTGCTTCTA
pycA	Forward	GCGTTAGGTGGAAACGAAAG
	Reverse	CGCGTCCAAGTTTATGGAAT
tpi	Forward	GCCCAGTAGCACTTAGCGAC
	Reverse	CCGAAACCGTCAAGAATGAT
